# Tislelizumab plus chemotherapy is more cost-effective than chemotherapy alone as first-line therapy for advanced non-squamous non-small cell lung cancer

**DOI:** 10.3389/fpubh.2023.1009920

**Published:** 2023-01-30

**Authors:** Xueyan Liang, Xiaoyu Chen, Huijuan Li, Yan Li

**Affiliations:** Department of Pharmacy, Guangxi Academy of Medical Sciences and the People's Hospital of Guangxi Zhuang Autonomous Region, Nanning, China

**Keywords:** tislelizumab, chemotherapy, non-small cell lung cancer, partitioned survival model, cost-effectiveness

## Abstract

**Background and objective:**

Tislelizumab is a programmed cell death protein-1 (PD-1) inhibitor. Tislelizumab plus chemotherapy as first-line option for advanced non-squamous non-small cell lung cancer (NSCLC), compared with chemotherapy alone, resulted in significantly prolonged survival outcomes; however, evidence regarding its relative efficacy and cost is lacking. We aimed to evaluate the cost-effectiveness of tislelizumab plus chemotherapy compared with that of chemotherapy alone, from the health care perspective in China.

**Methods:**

A partitioned survival model (PSM) was used for this study. The survival data were obtained from the RATIONALE 304 trial. Cost-effectiveness was defined as incremental cost-effectiveness ratio (ICER) less than the willingness to pay (WTP) threshold. Incremental net health benefits (INHB), incremental net monetary benefits (INMB), and subgroup analyses were also assessed. Sensitivity analyses were further established to assess the model stability.

**Results:**

Compared with chemotherapy alone, tislelizumab plus chemotherapy increased by 0.64 quality-adjusted life-years (QALYs) and 1.48 life-years, and yielded an increase of $16,631 in cost per patient. The INMB and INHB were $7,510 and 0.20 QALYs at a WTP threshold of $38,017/QALY, respectively. The ICER was $26,162/QALY. The outcomes were most sensitive to the HR of OS for tislelizumab plus chemotherapy arm. The probability of tislelizumab plus chemotherapy being considered cost-effective was 87.66% and >50% in most of the subgroups at the WTP threshold of $38,017/QALY. At the WTP threshold of $86,376/QALY, the probability achieved 99.81%. Furthermore, the probability of tislelizumab plus chemotherapy being considered cost-effective in subgroups of patients with liver metastases and PD–L1 expression ≥50% were 90.61 and 94.35%, respectively.

**Conclusion:**

Tislelizumab plus chemotherapy is likely to be cost-effective as a first-line treatment for advanced non-squamous NSCLC in China.

## Introduction

Lung cancer is on the rise worldwide, and the most common cause of annual cancer deaths (1). Non-small cell lung cancer (NSCLC) comprises ~85% of all lung cancer cases (2), and non-squamous cell carcinomas constitute 70–75% of NSCLC cases (3). Platinum-based chemotherapy (PBC) had always been a standard first-line option for patients with advanced NSCLC in the past (4, 5). However, these interventions were associated with < 1-year median overall survival (OS) (6). Targeted agents, however, benefit some patients and were reportedly associated with a median OS of 31.8–38.6 months among few patients with epidermal growth factor receptor (EGFR), anaplastic lymphoma kinase (ALK), or other sensitive driver gene mutations (7, 8). Resistance to targeted agents is an unavoidable problem. Considering the prevalence and dismal outcomes of advanced NSCLC, new interventions and combinations to improve survival are urgently needed.

Tislelizumab a programmed death ligand 1 (PD-L1)/programmed death ligand 2 (PD-L2)–mediated cell signaling antagonist, can increase cytokine production and restore T-cell activation, resulting in immune-mediated tumor cell death (9). Tislelizumab has revealed encouraging efficacy for advanced NSCLC (10, 11). In combination treatment with chemotherapy, tislelizumab has also shown significantly improved survival and durable clinical responses (12, 13). Recently, a RATIONALE 304 (14) trial evaluated tislelizumab plus pemetrexed and PBC as first-line therapy for patients with locally advanced non-squamous NSCLC in China. This trial illustrated that tislelizumab plus chemotherapy significantly prolonged progression-free survival (PFS), and resulted in higher response rates and longer response duration when compared with chemotherapy alone. The safety evaluation of tislelizumab plus chemotherapy corresponded with the known risks of each study treatment component, and no new safety signals were identified (11, 14). In June 2020, tislelizumab plus chemotherapy received approval as first-line treatment for patients with advanced non-squamous NSCLC in China (11).

Although these results are encouraging, the relatively higher cost of the combination treatment (tislelizumab plus chemotherapy) compared with chemotherapy alone necessitated the establishment of an urgent pharmacoeconomic evaluation. Accordingly, from the health care perspective in China, this study aimed to conduct a cost-effectiveness analysis of tislelizumab plus chemotherapy vs. chemotherapy alone as the first-line treatment in patients with advanced non-squamous NSCLC.

## Materials and methods

### Patients and intervention

We performed this study following the Consolidated Health Economic Evaluation Reporting Standards (CHEERS) reporting (15). The target patients were selected from the patients with advanced non-squamous NSCLC from the RATIONALE 304 trial (14). In this trial, included patients were at least 18 years old with histologically confirmed locally advanced (stage IIIB) or metastatic (stage IV) non-squamous NSCLC without known EGFR sensitizing mutations or ALK rearrangements. Enrolled patients were randomized to receive chemotherapy [carboplatin area under the curve 5 or cisplatin (75 mg/m^2^) in combination with pemetrexed (500 mg/m^2^)] only, or receive tislelizumab 200 mg plus chemotherapy. Both groups received intravenous injections once every 3 weeks. According to the National Comprehensive Cancer Network (NCCN) and Chinese Society of Clinical Oncology (CSCO) guidelines and considering the safety profile of tislelizumab or pemetrexed, the maintenance of tislelizumab plus pemetrexed or pemetrexed were continued until clinical progression.

### Partitioned survival model

A partitioned survival model (PSM) with 3 mutually exclusive health states was constructed for an initial decision regarding treatment with tislelizumab plus chemotherapy or with chemotherapy alone in this economic evaluation; PFS, progressed disease (PD), and death (16). The longest simulation period was 15 years, which simulated more than 98% of the deaths in both treatment groups, and the cycle length was 1 week. In the three health states, OS was partitioned into “alive with PFS” and “alive and with PD.” The proportion of patients alive at cycle t (1-week cycle) was estimated by the area under the OS curve, and the proportion alive with PFS was estimated by the area under the PFS curve. The proportion alive and with PD was estimated by the difference between the OS and PFS curves. In the model, the proportions of OS and PFS events were established according to the clinical data from the RATIONALE 304 trial (14).

### Clinical data inputs

Both OS and PFS survival curves obtained from the RATIONALE 304 trial were established following the algorithm created by Guyot et al. (17). We used the GetData Graph Digitizer version 2.26 to obtain the time-to-survival data points for Kaplan-Meier (K-M) survival curves of OS and PFS (18). Then, we calculated these data points to fit the parametric survival functions, including Exponential, Weibull, Gamma, Lognormal, Gompertz, Generalized gamma distributions, and Log-logistic. Next, using the Akaike Information Criterion (AIC) and Bayesian Information Criterion (BIC), lower values indicated better fit of the selected model for the reconstructed K-M survival curves. The parametric model results of the tislelizumab plus chemotherapy or chemotherapy groups are summarized in Table 1, and the results of goodness-of-fit are listed in Supplementary Table 1. Lognormal was selected to fit the OS K-M curves of tislelizumab plus chemotherapy or chemotherapy, and Weibull was selected to fit the PFS K-M curves (Supplementary Figure 1). Virtual patient-level data comprised event and censor times and were equal in number to the initial number at risk, which closely reproduced the digitized K-M curves. Considering that the primary trial lacked detailed information on the subsequent treatment strategies, the CSCO and the NCCN guidelines were used for subsequent treatment strategies in patients after disease progression in our analysis (Table 1). The key clinical input data are shown in Table 1 (14, 19–29).

**Table 1:** Key model inputs.

**Parameter**	**Value (95% CI)**	**Distribution**	**Source**
**Clinical input**			
**Survival model for tislelizumab plus chemotherapy**			
Lognormal model for OS^a^	μ = 4.6498 σ = 0.9724	ND	(14)
Weibull model for PFS^a^	γ = 1.5079 λ = 0.0028	ND	(14)
**Survival model for chemotherapy**			
Lognormal model for OS^a^	μ = 4.44785 σ = 1.17556	ND	(14)
Weibull model for PFS^a^	γ = 1.5206 λ = 0.0044	ND	(14)
**Cost input**			
**Drug costs per 1 mg**			
Tislelizumab	2.16 (1.73 to 2.59)	Gamma	Local database
Carboplatin	0.12 (0.08 to 0.16)	Gamma	Local database
Pemetrexed	1.17 (0.22 to 3.19)	Gamma	Local database
Nivolumab	15.44 (13.79 to 17.09)	Gamma	Local database
Docetaxel	1.61 (0.73 to 2.25)	Gamma	Local database
Second-line treatment in tislelizumab plus chemotherapy arm per cycle	430.79 (344.63 to 516.95)	Gamma	(14); Local database
Second-line treatment in chemotherapy arm per cycle	648.59 (518.87 to 778.31)	Gamma	(14); Local database
Cost of terminal care per patient^b^	2,464.50 (1,848.38 to 3,080.63)	Gamma	(19)
**Disease costs per cycle**			
Patients with PFS^c^	175.50 (131.63 to 219.38)	Gamma	(19)
Patients with PD^c^	523.50 (392.63 to 654.38)	Gamma	(19)
**Cost of managing AEs (grade** ≥**3)**			
Tislelizumab plus chemotherapy	4,326 (34,618 to 5,191)	Gamma	(20–22)
Chemotherapy	3,193 (2,554 to 3,832)	Gamma	(20–22)
Supportive care per cycle^d^	72 (58 to 86)	Gamma	(23)
Cost of drug administration per unit	19.11 (15.288 to 22.932)	Gamma	(24)
**Health utilities**			
**Disease status utility per year**			
Utility of PFS	0.804 (0.64 to 0.96)	Beta	(25)
Utility of PD	0.321 (0.26 to 0.39)	Beta	(25)
Death	0	NA	
**Disutility due to AEs**			
Tislelizumab plus chemotherapy	0.202 (0.162 to 0.242)	Beta	(25–27)
Chemotherapy	0.157 (0.126 to 0.188)	Beta	(25–27)
Body surface area, m^2^	1.8 (1.44 to 2.16)	Normal	(28, 29)
Body weight, kg	65 (50 to 90)	Normal	(28, 29)
Creatinine clearance rate, ml/min/1.73 m^2^	90 (80 to 12)	Normal	(29)

ND, not determined; OS, overall survival; PD, progressed disease; PFS, progression-free survival; AE, adverse event.

a Only expected values are presented for these survival model parameters.

b Overall total cost per patient regardless of treatment duration.

c These costs were assumed to be continued until the health state transitioned.

d The cost of routine follow-up included the cost of outpatient physician visit, laboratory tests and examinations.

### Cost inputs

The following direct medical costs were considered: the cost of acquiring drugs, the cost associated with the patient's health state, cost of supportive care, grade ≥3 adverse events (AEs)-related costs, and the cost of terminal care (Table 1). The costs were obtained in Chinese Yuan and were exchanged to US dollars, with the exchange rate of 2021 (1 US dollar = 6.37 Chinese Yuan) (30). Costs of the drugs were collected from the standard fee database. To calculate the median dosage of chemotherapy and immune checkpoint inhibitors, we assumed the average body surface area (BSA), creatinine clearance rate (Ccr), and weight to be 1.80 m^2^, 90 ml/min/1.73 m^2^, and 65 kg, respectively (28, 29). Additionally, the costs associated with the management of grade ≥ 3 AEs were calculated by multiplying the rates provided in the randomized controlled trial, and the cost of AEs management was derived from the published literature (Supplementary Table 2) (20–22, 25–27). Considering that the primary trial lacked detailed information on the subsequent treatment strategies, we adopted standard treatment strategies that were recommended by the CSCO and NCCN guidelines in our analysis (Table 1). The costs associated with disease state, subsequent supportive care, drug administration, and terminal care were obtained from the published literature (Table 1).

### Utility inputs

Health utility scores were assigned in the range of death (0) to perfect health (1). Considering the RATIONALE 304 trial did not provide the PD and PFS health utilities; thus, these were obtained from the published literature. The PD and PFS health utilities associated with advanced NSCLC were 0.804 (25) and 0.321 (25), respectively. The disutility values related to grade ≥3 AEs are presented in Supplementary Table 2 (20–22, 25–27).

### Base-case analysis

The incremental cost-effectiveness ratios (ICERs) were calculated to assess the incremental cost per additional quality-adjusted life-year (QALY) gained. Considering the imbalanced economic development among different socioeconomic regions in China, we calculated ICERs with two willingness to pay (WTP) thresholds: three times the per capita gross domestic product (GDP) value of China in 2021 (USD 38,017/QALY) for general regions and three times of the per capita GDP value of Beijing city in 2021 (USD 86,376/QALY) for affluent regions (31). A 5% discount rate annually was applied for utility outcomes and costs (32). The incremental net health benefit (INHB) and incremental monetary benefit (INMB) were also calculated based on the following formulas:


$$\text{INMB}(\text{λ})=(\mu_{\text{Etc}}-\mu_{\text{Ec}})-\frac{\mu_{\text{Ctc}}-\mu_{\text{Cc}}}{\text{λ}}=\;\Delta E-\Delta C/\text{λ}$$

and


$$\text{INMB}(\text{λ})=(\mu_{\text{Etc}}-\mu_{\text{Ec}})\times \text{λ}-(\mu_{\text{Ctc}}-\mu_{\text{Cc}})=\;\Delta E\times \text{λ}-\Delta C,$$

where μ_Ctc_, μ_Cc_, μ_Etc_, and μ_Ec_ were the cost and effectiveness of tislelizumab plus chemotherapy or chemotherapy, respectively, and λ was the WTP threshold (33). If the ICER of tislelizumab plus chemotherapy vs. chemotherapy is below the WTP threshold, the tislelizumab plus chemotherapy regimen is generally considered to be cost-effective.

### Sensitivity analysis

Sensitivity analyses were performed for model input parameters to evaluate the robustness of the model. Through one-way deterministic sensitivity analysis, the impact of uncertainty of different variables on the ICER was discussed. The uncertainty of each variable was adjusted within 95% confidence intervals (CIs) reported in the literature or assuming reasonable ranges of the fundamental parameters (±25%) variation from the fundamental parameters (Table 1). In addition, a probabilistic sensitivity analysis was performed by Monte Carlo simulations with 10,000 iterations. All input parameters were sampled simultaneously on a basis of an appropriate distribution. The cost parameters were sampled from the Gamma distribution, and the health utility parameters were sampled from the Beta distribution. Based on the data from 10,000 iterations, the probabilistic sensitivity analyses are presented as cost-effectiveness acceptability curve to describe the likelihood that tislelizumab plus chemotherapy or chemotherapy would be valuable at different WTP levels for/QALYs gains.

### Subgroup analysis

To investigate the uncertainty of outcomes caused by patients with different characteristics, subgroup analysis was also performed. Subgroup analysis was performed for the different subgroups that were obtained from the RATIONALE 304 (14) by varying the HRs for PFS. Statistical analyses in this study were programmed in R with hesim and heemod packages, version 4.0.5.

## Results

### Base-case analysis

For the total population with advanced non-squamous NSCLC, tislelizumab plus chemotherapy resulted in an increase of 0.64 QALYs effectiveness and 1.48 overall life-years vs. chemotherapy alone, as well as an additional cost of $16,631. The corresponding ICER was $26,162/QALY. Moreover, at a $38,017/QALY WTP threshold compared with chemotherapy alone, the INHB and INMB of tislelizumab plus chemotherapy were 0.20 QALYs and $7,510, respectively (Table 2).

**Table 2:** Summary of cost and outcome results in the base-case analysis.

**Factor**	**Tislelizumab plus chemotherapy**	**Chemotherapy**	**Incremental change**
**Cost, $**			
Drug^a^	100,670	90,788	9,883
Non-drug^a^	9,683	2,934	6,748
Overall	110,353	93,722	16,631
**Life-years**			
Progression-free	1.15	0.61	0.54
Overall	4.41	2.93	1.48
QALYs	1.75	1.12	0.64
**ICERs, $**			
Per life-year	NA	NA	11,263
Per QALY	NA	NA	26,162
INHB, QALY, at threshold 38,017^a^	NA	NA	0.20
INMB, $, at threshold 38,017^a^	NA	NA	7,510

ICER, incremental cost-effectiveness ratio; INHB, incremental net health benefit; INMB, incremental net monetary benefit; NA, not applicable; QALYs, quality-adjusted life-years.

a Compared with chemotherapy alone.

### Sensitivity analysis

**Figure 1:**
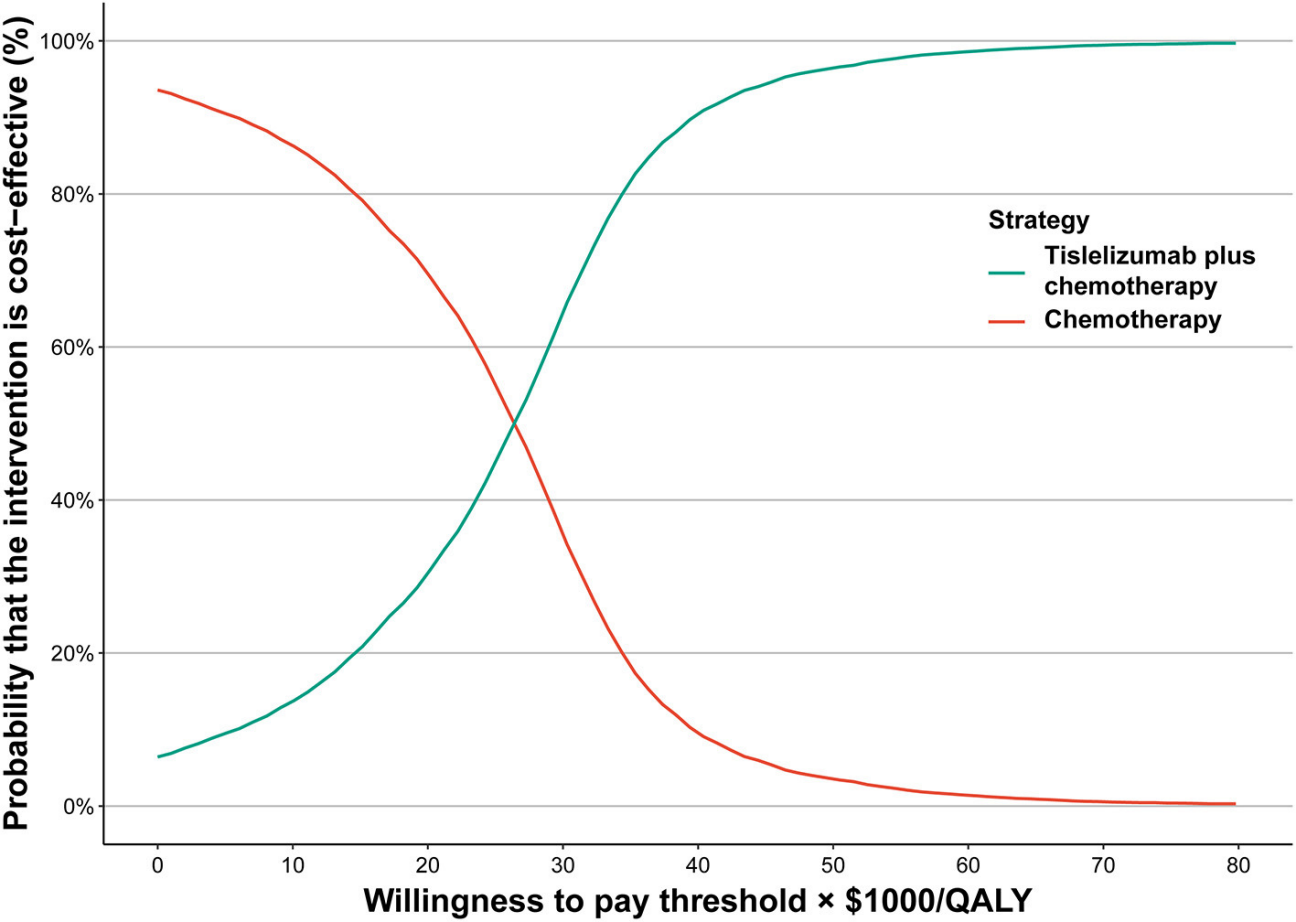
Acceptability curves of cost-effectiveness. QALY, quality-adjusted life-year.

In the cost-effectiveness acceptability curve for the whole population, at the WTP threshold of $38,017/QALY, the probability of tislelizumab plus chemotherapy being cost-effective was 87.66%, and for the WTP threshold of $86,376/QALY, the probability of tislelizumab plus chemotherapy being considered cost-effective was 99.81% (Figure 1).

The results of the one-way sensitivity analysis illustrated that the HR of OS for tislelizumab plus chemotherapy arm influenced the outcomes to be most sensitive. The remaining parameters were only moderately or marginally associated with the model (Supplementary Figure 2).

### Subgroup analysis

**Figure 2:**
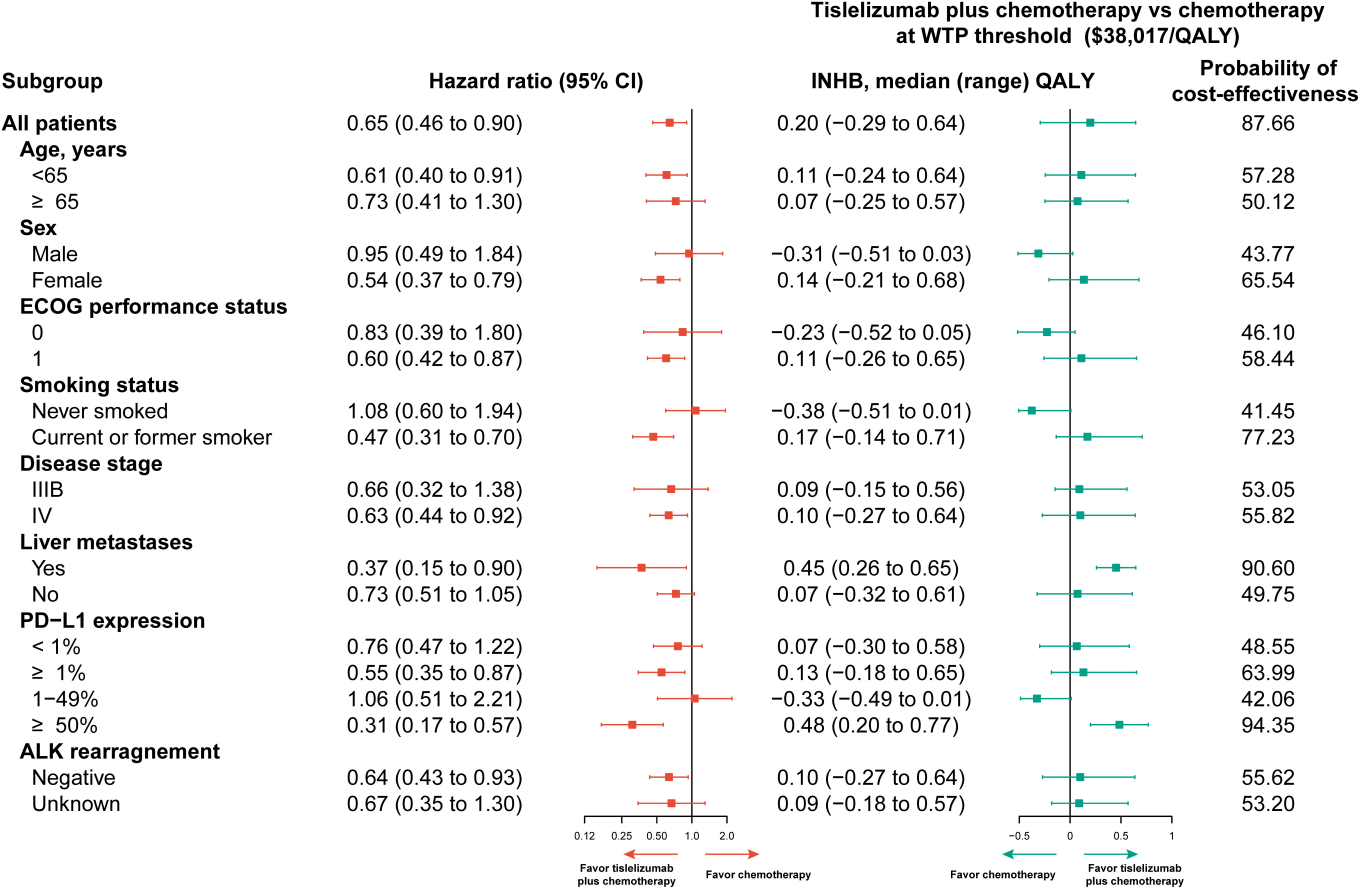
Subgroup analyses. Subgroup analyses. CI, confidence interval; ECOG, Eastern Cooperative Oncology Group; ICER, incremental cost-effectiveness ratio; INHB, incremental net health benefits; PD-L1, programmed cell death ligand 1; QALY, quality-adjusted life-year; WTP, willingness to pay.

This subgroup analysis was established by varying the HRs for PFS. For most subgroups, tislelizumab plus chemotherapy yielded better in lowering the risk of death. There was an overall trend that subgroups with better survival advantages associated with a higher probability to be cost-effective. The probability of tislelizumab plus chemotherapy being considered cost-effective was > 50% in most subgroups at the WTP threshold of $38,017/QALY, including patients who less or more than 65 years of age, female patients, patients with Eastern Cooperative Oncology Group (ECOG) performance status 1, and patients who are/were current or former smoker. Furthermore, the probability of tislelizumab plus chemotherapy being considered cost-effective in subgroups of patients with liver metastases and PD–L1 expression ≥50% was 90.06 and 94.35%, respectively (Figure 2).

## Discussion

In this study we performed cost-effectiveness analyses of tislelizumab plus chemotherapy vs. chemotherapy alone for advanced non-squamous NSCLC as first-line treatment. According to the RATIONALE 304 trial, addition of tislelizumab to chemotherapy significantly prolonged PFS when compared with chemotherapy alone, and resulted in higher response rates and longer response duration for patients with advanced non-squamous NSCLC (14). However, based on a comparison of tislelizumab plus chemotherapy and chemotherapy alone, it is uncertain which is a preferable option for physicians and patients; hence, conducting an cost-effectiveness analysis to evaluate the value of tislelizumab plus chemotherapy in terms of both efficacy and cost is urgently needed.

In this study, tislelizumab plus chemotherapy was related to an increase of 0.64 QALYs and an additional cost of $16,631. The calculated corresponding ICER was $26,162/QALY. For the whole population, at the WTP threshold of $38,017/QALY, the probability of tislelizumab plus chemotherapy being considered cost-effective was 87.66%, and for the WTP threshold of $86,376/QALY, the probability of tislelizumab plus chemotherapy being considered cost-effective was 99.81%. One-way sensitivity analyses revealed that the HR of OS for tislelizumab plus chemotherapy arm was the most sensitive factor for the ICER. The model is robust according to the sensitivity analysis results. An ICER of $26,162/QALY for tislelizumab plus chemotherapy was calculated in this study compared with chemotherapy alone. The ICER is less than the WTP threshold of $38,017/QALY. Hence, tislelizumab plus chemotherapy is likely to be considered cost-effective as first-line therapy for patients with advanced non-squamous NSCLC, compared with chemotherapy alone.

Previously published study evaluated the cost-effectiveness of tislelizumab plus chemotherapy for locally advanced or metastatic non-squamous NSCLC (34). It revealed that tislelizumab plus chemotherapy was related to an increase of 0.99 QALYs and an additional cost of $28,749, and yielded corresponding ICER was $28,749/QALY. Compared with previous study, several differences were noted in this study. First, in this study, we adopted PSM to evaluate the cost-effectiveness of tislelizumab plus chemotherapy for NSCLC, and Markov models was used in previous published study. In a PSM, health state occupancy is estimated directly from the area under the relevant survival curve. PSMs differ from state transition models (STM) such as Markov models, as they do not include a structural link between intermediate clinical endpoints (e.g., disease progression) and survival. PSMs directly consider clinical trial endpoints and can be developed without access to individual patient data. On the other hand, PSMs and STMs can produce substantively different survival extrapolations and extrapolations from STMs are heavily influenced by specification of the underlying survival models (35). In a PSM, OS extrapolation reflects only the OS evidence and not PFS, whereas in a STM, OS extrapolation is influenced by the model structure and each transition probability estimate (35). Second, in this study, we obtained the price of drugs from the local database of several hospital, and the public hospitals in China implemented a policy that the selling price of drugs was in accordance with the purchasing price of drugs. The cost of drugs can be reflected as the real situation in China. Third, the maintenance of tislelizumab plus chemotherapy or chemotherapy was not provided in previously published study. According to the RATIONALE 304 trial, patients in tislelizumab plus chemotherapy group followed by maintenance tislelizumab plus pemetrexed treatment, and in chemotherapy group followed by maintenance pemetrexed treatment. This may result in the variance of the cost compared with the truth. Fourth, in this study, we also considered the disease management costs. Disease management costs were incurred in both PFS and PD. The common costs included blood tests, chest X-ray, abdominal CT scan, radiation therapy, home healthcare, nurse, medical specialists, and hospital fees. In general, PD was associated with more hospital fees than PFS, especially in emergency department and ICU. Lastly, in this study, we also calculated INHB and INMB, in a resource-constrained health care system, health care costs really represent the health outcomes for other patients with competing claims on health care resources; therefore, decisions based on economic evaluation are really about identifying the alternative which offers the greatest INHB or INMB overall (36, 37). Our result is consistent with that of a previous study in which tislelizumab being cost-effective vs. docetaxel for pretreated advanced NSCLC in China (38). The previous study revealed that the corresponding ICER was $18,122/QALY, less than the WTP threshold of three times GDP per capita in China, and the probability of tislelizumab being considered cost-effective was 96.79% (38). In addition, the previous study also revealed that tislelizumab was not to be considered cost-effective before national negotiation of China in 2020 (38). Actually, great efforts have been made by the National Healthcare Security Administration (NHSA) in China to negotiate the prices of drugs with pharmaceutical companies, and the prices of many anticancer drugs including immune checkpoint inhibitors have dropped by 30–70% in return (39). For example, in March 2021, the price of camrelizumab dropped from $14.35/mg to $2.12/mg as it was successfully negotiated and incorporated into the National Reimbursement Drug List (NRDL) in China (40). For this reason, a trend of rising price for tislelizumab is unlikely. Therefore, our findings revealed that first-line use of tislelizumab plus chemotherapy can be sufficient to support as a cost-effective option for patients with advanced non-squamous NSCLC.

The advantages of this research are worth emphasizing. First, this is the first evaluation of the cost-effectiveness of tislelizumab plus chemotherapy compared with chemotherapy alone for advanced non-squamous NSCLC. Moreover, a PSM, which has variables included to sufficiently explore the cost-effectiveness of immunotherapies, was established. The PSM does not require assumptions about the transition between different health states, but allows researchers to directly classify patients into different health states according to the K-M curve. Second, the patients evaluated in the trial were Chinese, which rules out the influence of racial difference. Last, the present study evaluated the economic outcomes of 18 subgroups researched by the RATIONALE 304 trial (14). Physicians could benefit from the economic data based on subgroup information to make personalized treatment decisions.

This study has some limitations. First, clinical data in the model of this study on the basis of the outcomes of the RATIONALE 304 trial. Therefore, any bias in the trial could affect the cost and effectiveness results. Second, healthcare costs were mainly derived from previous literature. However, sensitivity analyses revealed that cost-related parameters had negligible impact on the economic outcomes. The drug costs were calculated according to the latest prices, which may represent the real-world data more accurately. Lastly, health outcomes were evaluated based on the goodness-of-fit of the RATIONALE 304 trial were assumed by fitting data to the published K–M OS and PFS data, which could lead to uncertainty in the model outputs, and this may underestimate survival benefit of tislelizumab plus chemotherapy arm. However, we are sure that the model chosen in this study is the most appropriate models to fit the K–M OS and PFS data.

## Conclusion

In conclusion, from the health care perspective in China, tislelizumab plus chemotherapy as first-line treatment being considered cost-effective for patients with non-squamous NSCLC vs. chemotherapy alone. Personalized treatment based on subgroup analysis may improve economic outcomes, which may help clinicians in deciding appropriate treatment for advanced non-squamous NSCLC.

## Data availability statement

The original contributions presented in the study are included in the article/Supplementary material, further inquiries can be directed to the corresponding author.

## Author contributions

XL and YL: gathering and analyzing all data, drafting, and statistical analysis. XL, YL, and XC: concept and design. XC: funding. HL and XL: technical and material support and supervision. All authors: critical revision of the manuscript and data interpretation. All authors contributed to the article and approved the submitted version.

## References

[1] Siegel RL Miller KD Jemal A. Cancer statistics, 2020. CA Cancer J Clin. (2020) 70:7–30. 10.3322/caac.21590 31912902

[2] Herbst RS Morgensztern D Boshoff C. The biology and management of non-small cell lung cancer. Nature. (2018) 553:446–54. 10.1038/nature25183 29364287

[3] Siegel RL Miller KD Fuchs HE Jemal A. Cancer statistics, 2022. CA Cancer J Clin. (2022) 72:7–33. 10.3322/caac.21708 35020204

[4] Polanski J Jankowska-Polanska B Rosinczuk J Chabowski M Szymanska-Chabowska A. Quality of life of patients with lung cancer. Onco Targets Ther. (2016) 9:1023–8. 10.2147/OTT.S100685 27013895 PMC4778772

[5] National Comprehensive Cancer Network. Nccn Clinical Practice Guidelines in Oncology: Non-Small Cell Lung Cancer, Version 4. Available online at: https://www.nccn.org/guidelines/ (accessed May 23, 2022).

[6] Arbour KC Riely GJ. Systemic therapy for locally advanced and metastatic non-small cell lung cancer: a review. JAMA. (2019) 322:764–74. 10.1001/jama.2019.11058 31454018

[7] Fukuoka M Wu YL Thongprasert S Sunpaweravong P Leong SS Sriuranpong V. Biomarker analyses and final overall survival results from a phase Iii, randomized, open-label, first-line study of gefitinib vs. carboplatin/paclitaxel in clinically selected patients with advanced non-small-cell lung cancer in Asia (Ipass). J Clin Oncol. (2011) 29:2866–74. 10.1200/JCO.2010.33.4235 21670455

[8] Gridelli C Ciardiello F Gallo C Feld R Butts C Gebbia V. First-line erlotinib followed by second-line cisplatin-gemcitabine chemotherapy in advanced non-small-cell lung cancer: the torch randomized trial. J Clin Oncol. (2012) 30:3002–11. 10.1200/JCO.2011.41.2056 22778317

[9] Zhang T Song X Xu L Ma J Zhang Y Gong W. The binding of an Anti-Pd-1 antibody to Fcγrι has a profound impact on its biological functions. Cancer Immunol Immunother. (2018) 67:1079–90. 10.1007/s00262-018-2160-x 29687231 PMC6006217

[10] Shen L Guo J Zhang Q Pan H Yuan Y Bai Y. Tislelizumab in Chinese patients with advanced solid tumors: an open-label, non-comparative, phase 1/2 study. J Immunother Cancer. (2020) 8:1. 10.1136/jitc-2019-000437 32561638 PMC7304812

[11] Liu SY Wu YL. Tislelizumab: an investigational Anti-Pd-1 antibody for the treatment of advanced non-small cell lung cancer (Nsclc). Expert Opin Investig Drugs. (2020) 29:1355–64. 10.1080/13543784.2020.1833857 33044117

[12] Wang Z Zhao J Ma Z Cui J Shu Y Liu Z. A phase 2 study of tislelizumab in combination with platinum-based chemotherapy as first-line treatment for advanced lung cancer in chinese patients. Lung Cancer. (2020) 147:259–68. 10.1016/j.lungcan.2020.06.007 32769013

[13] Wang J Lu S Yu X Hu Y Sun Y Wang Z. Tislelizumab plus chemotherapy vs chemotherapy alone as first-line treatment for advanced squamous non-small-cell lung cancer: a phase 3 randomized clinical trial. JAMA Oncol. (2021) 7:709–17. 10.1001/jamaoncol.2021.0366 33792623 PMC8017481

[14] Lu S Wang J Yu Y Yu X Hu Y Ai X. Tislelizumab plus chemotherapy as first-line treatment for locally advanced or metastatic nonsquamous nsclc (rationale 304): a randomized phase 3 trial. J Thorac Oncol. (2021) 16:1512–22. 10.1016/j.jtho.2021.05.005 34033975

[15] Husereau D Drummond M Augustovski F de Bekker-Grob E Briggs AH Carswell C. Consolidated health economic evaluation reporting standards 2022 (Cheers 2022) Statement: updated reporting guidance for health economic evaluations. Value Health. (2022) 25:3–9. 10.1016/j.jval.2021.11.1351 35031096

[16] Williams C Lewsey JD Mackay DF Briggs AH. Estimation of survival probabilities for use in cost-effectiveness analyses: a comparison of a multi-state modeling survival analysis approach with partitioned survival and markov decision-analytic modeling. Med Decis Making. (2017) 37:427–39. 10.1177/0272989X16670617 27698003 PMC5424853

[17] Guyot P Ades AE Ouwens MJ Welton NJ. Enhanced secondary analysis of survival data: reconstructing the data from published Kaplan-Meier survival curves. BMC Med Res Methodol. (2012) 12:9. 10.1186/1471-2288-12-9 22297116 PMC3313891

[18] Getdata Graph Digitizer. Available online at: http://getdata-graph-digitizer.com (accessed June 09, 2022).

[19] Jiang Y Wang X. Cost-effectiveness analysis of pembrolizumab plus standard chemotherapy vs. chemotherapy alone for first-line treatment of metastatic non-squamous non–small-cell lung cancer in China. Eur J Hosp Pharm. (2022) 29:139–44. 10.1136/ejhpharm-2020-002208 32737070 PMC9047884

[20] Wu B Dong B Xu Y Zhang Q Shen J Chen H. Economic evaluation of first-line treatments for metastatic renal cell carcinoma: a cost-effectiveness analysis in a health resource-limited setting. PLoS ONE. (2012) 7:e32530. 10.1371/journal.pone.0032530 22412884 PMC3297611

[21] Wong W Yim YM Kim A Cloutier M Gauthier-Loiselle M Gagnon-Sanschagrin P. Assessment of costs associated with adverse events in patients with cancer. PLoS ONE. (2018) 13:e0196007. 10.1371/journal.pone.0196007 29652926 PMC5898735

[22] Zheng H Xie L Zhan M Wen F Xu T Li Q. Cost-effectiveness analysis of the addition of bevacizumab to chemotherapy as induction and maintenance therapy for metastatic non-squamous non-small-cell lung cancer. Clin Transl Oncol. (2018) 20:286–93. 10.1007/s12094-017-1715-1 28785913

[23] Gu X Zhang Q Chu YB Zhao YY Zhang YJ Kuo D. Cost-effectiveness of afatinib, gefitinib, erlotinib and pemetrexed-based chemotherapy as first-line treatments for advanced non-small cell lung cancer in China. Lung Cancer. (2019) 127:84–9. 10.1016/j.lungcan.2018.11.029 30642557

[24] Wu B Zhang Q Sun J. Cost-effectiveness of nivolumab plus ipilimumab as first-line therapy in advanced renal-cell carcinoma. J Immunother Cancer. (2018) 6:124. 10.1186/s40425-018-0440-9 30458884 PMC6247499

[25] Nafees B Lloyd AJ Dewilde S Rajan N Lorenzo M. Health state utilities in non-small cell lung cancer: an international study. Asia Pac J Clin Oncol. (2017) 13:e195–203. 10.1111/ajco.12477 26990789

[26] Freeman K Connock M Cummins E Gurung T Taylor-Phillips S Court R. Fluorouracil plasma monitoring: systematic review and economic evaluation of the My5-Fu assay for guiding dose adjustment in patients receiving fluorouracil chemotherapy by continuous infusion. Health Technol Assess. (2015) 19:1–321. 10.3310/hta19910 26542268 PMC4781223

[27] Konidaris G Paul E Kuznik A Keeping S Chen CI Sasane M. Assessing the value of cemiplimab for adults with advanced cutaneous squamous cell carcinoma: a cost-effectiveness analysis. Value Health. (2021) 24:377–87. 10.1016/j.jval.2020.09.014 33641772

[28] Wu B Chen H Shen J Ye M. Cost-effectiveness of adding rh-endostatin to first-line chemotherapy in patients with advanced non-small-cell lung cancer in China. Clin Ther. (2011) 33:1446–55. 10.1016/j.clinthera.2011.09.016 21992806

[29] Zhu C Xing XX Wu B Liang G Han G Lin CX. Cost-effectiveness analysis of camrelizumab plus chemotherapy vs. chemotherapy alone as the first-line treatment in patients with Iiib-Iv non-squamous non-small cell lung cancer (Nsclc) without Egfr and Alk alteration from a perspective of health—care system in China. Front Pharmacol. (2021) 12:735536. 10.3389/fphar.2021.735536 35002693 PMC8740086

[30] China Bo. Foreign Exchange Rate. Available online at: https://www.boc.cn/sourcedb/whpj/ (accessed June 24, 2022).

[31] Eichler HG Kong SX Gerth WC Mavros P Jönsson B. Use of cost-effectiveness analysis in health-care resource allocation decision-making: how are cost-effectiveness thresholds expected to emerge? Value Health. (2004) 7:518–28. 10.1111/j.1524-4733.2004.75003.x 15367247

[32] Zhang Q Wu P He X Ding Y Shu Y. Cost-effectiveness analysis of camrelizumab vs. placebo added to chemotherapy as first-line therapy for advanced or metastatic esophageal squamous cell carcinoma in China. Front Oncol. (2021) 11:790373. 10.3389/fonc.2021.790373 34926306 PMC8671697

[33] Su D Wu B Shi L. Cost-effectiveness of atezolizumab plus bevacizumab vs sorafenib as first-line treatment of unresectable hepatocellular carcinoma. JAMA Netw Open. (2021) 4:e210037. 10.1001/jamanetworkopen.2021.0037 33625508 PMC7905498

[34] Luo X Zhou Z Zeng H Liu Q. The cost-effectiveness of tislelizumab plus chemotherapy for locally advanced or metastatic nonsquamous non-small cell lung cancer. Front Pharmacol. (2022) 13:935581. 10.3389/fphar.2022.935581 35935852 PMC9354466

[35] Woods BS Sideris E Palmer S Latimer N Soares M. Partitioned survival and state transition models for healthcare decision making in oncology: where are we now? Value Health. (2020) 23:1613–21. 10.1016/j.jval.2020.08.2094 33248517

[36] Culyer A McCabe C Briggs A Claxton K Buxton M Akehurst R. Searching for a threshold, not setting one: the role of the National Institute for Health and Clinical excellence. J Health Serv Res Policy. (2007) 12:56–8. 10.1258/135581907779497567 17244400

[37] McCabe C Claxton K Culyer AJ. The NICE cost-effectiveness threshold: what it is and what that means. Pharmacoeconomics. (2008) 26:733–44. 10.2165/00019053-200826090-00004 18767894

[38] Gong J Su D Shang J Xu S Tang L Sun Z. Cost-effectiveness of tislelizumab vs. docetaxel for previously treated advanced non-small-cell lung cancer in China. Front Pharmacol. (2022) 13:830380. 10.3389/fphar.2022.830380 35614942 PMC9124929

[39] Administration NHS. Notice of the National Healthcare Security Administration on Bringing 17 Kinds of Anticancer Drugs into the Category B of National Basic Medical Insurance, Industrial Injury Insurance and Maternity Insurance Drug List. Available online at: http://www.nhsa.gov.cn/art/2018/10/10/art_19_397.html (accessed April 12, 2022).

[40] Qiao L Zhou Z Zeng X Tan C. Cost-effectiveness of domestic pd-1 inhibitor camrelizumab combined with chemotherapy in the first-line treatment of advanced nonsquamous non-small-cell lung cancer in China. Front Pharmacol. (2021) 12:728440. 10.3389/fphar.2021.728440 34795580 PMC8593416

