# Supplementary material for: Tislelizumab plus chemotherapy is more cost-effective than chemotherapy alone as first-line therapy for advanced non-squamous non-small cell lung cancer
Source: Front Public Health. 2023 Jan 30;11:1009920. doi: 10.3389/fpubh.2023.1009920 (PMC9922748; doi:10.3389/fpubh.2023.1009920)
Supplement: Supplementary file 1 [file Data_Sheet_1.docx]

**Supplementary Content**

**Supplementary Figure 1.** Model Fitting Analysis

**Supplementary Figure 2.** Tornado Diagram of One-Way Sensitivity Analyses

**Supplementary Table 1.** Akaike Information Criterion and Bayesian Information Criterion Values from Each Survival Model

**Supplementary Table 2.** Associated Costs and Disutility of Grade ≥ 3 Treatment-Related Adverse Events

**Supplementary Figure 1.** Model Fitting Analysis

To obtain the best model fit, the following investigations were carried out using tislelizumab plus chemotherapy or chemotherapy as the model fit baseline, respectively. Based on values of AIC and BIC (Supplementary Table 1), Lognormal was used to fit the OS K-M curves of tislelizumab plus chemotherapy and chemotherapy. Weibull was used to fit the PFS K-M curves of tislelizumab plus chemotherapy and chemotherapy.

(A) Model-fitted versus original K-M curves for tislelizumab plus chemotherapy.


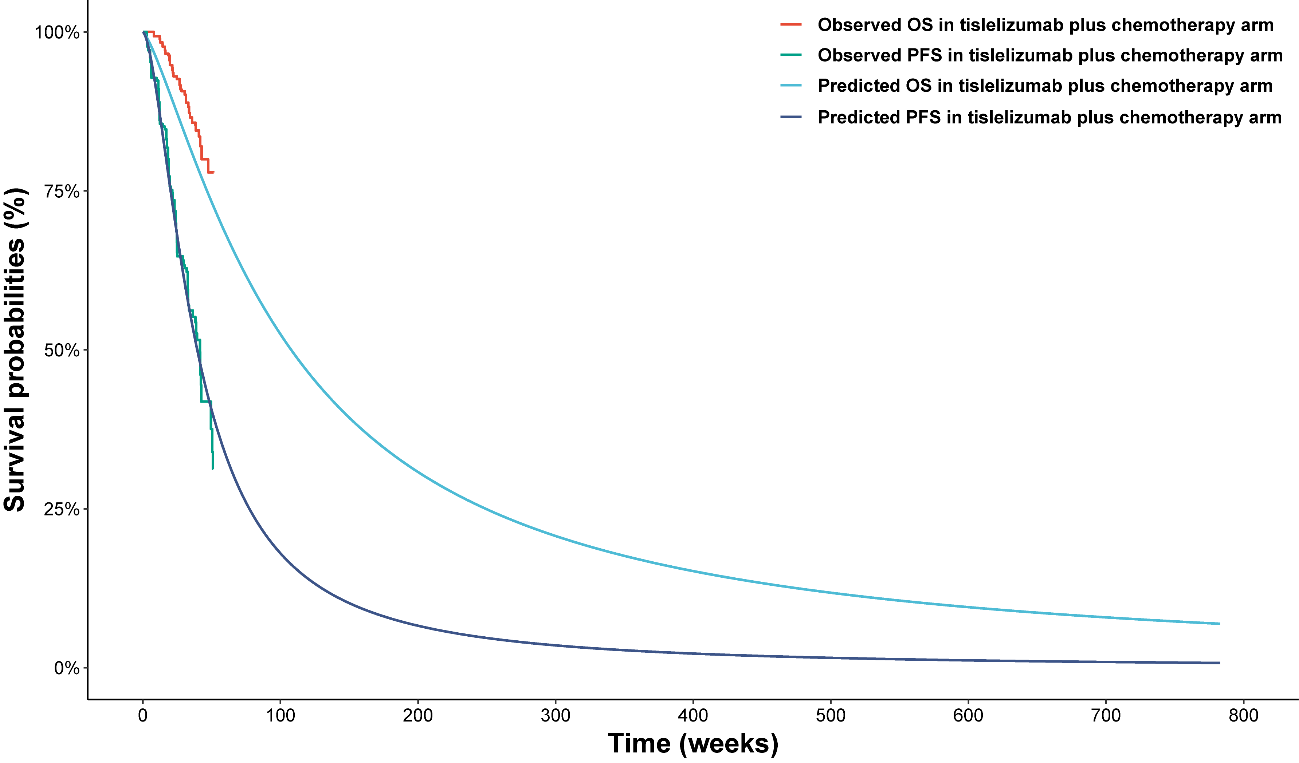


(B) Model-fitted versus original K-M curves for chemotherapy.


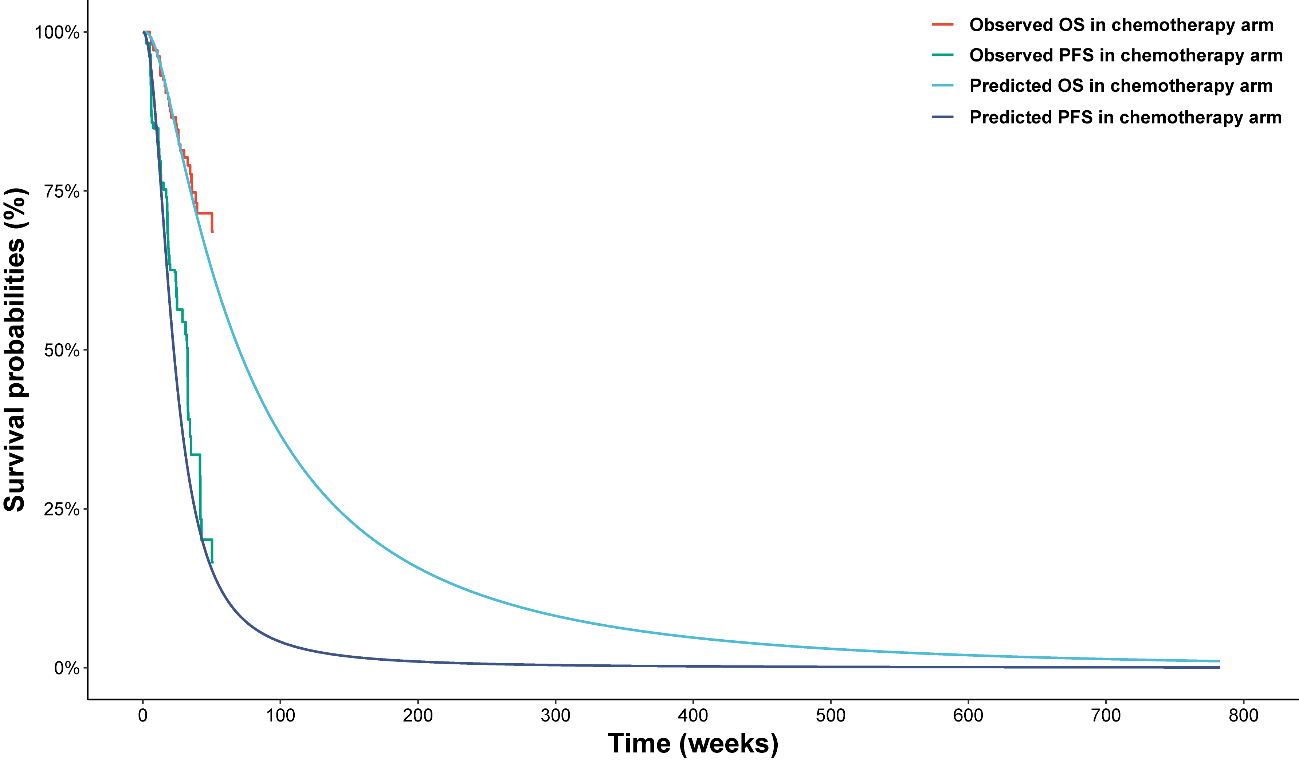


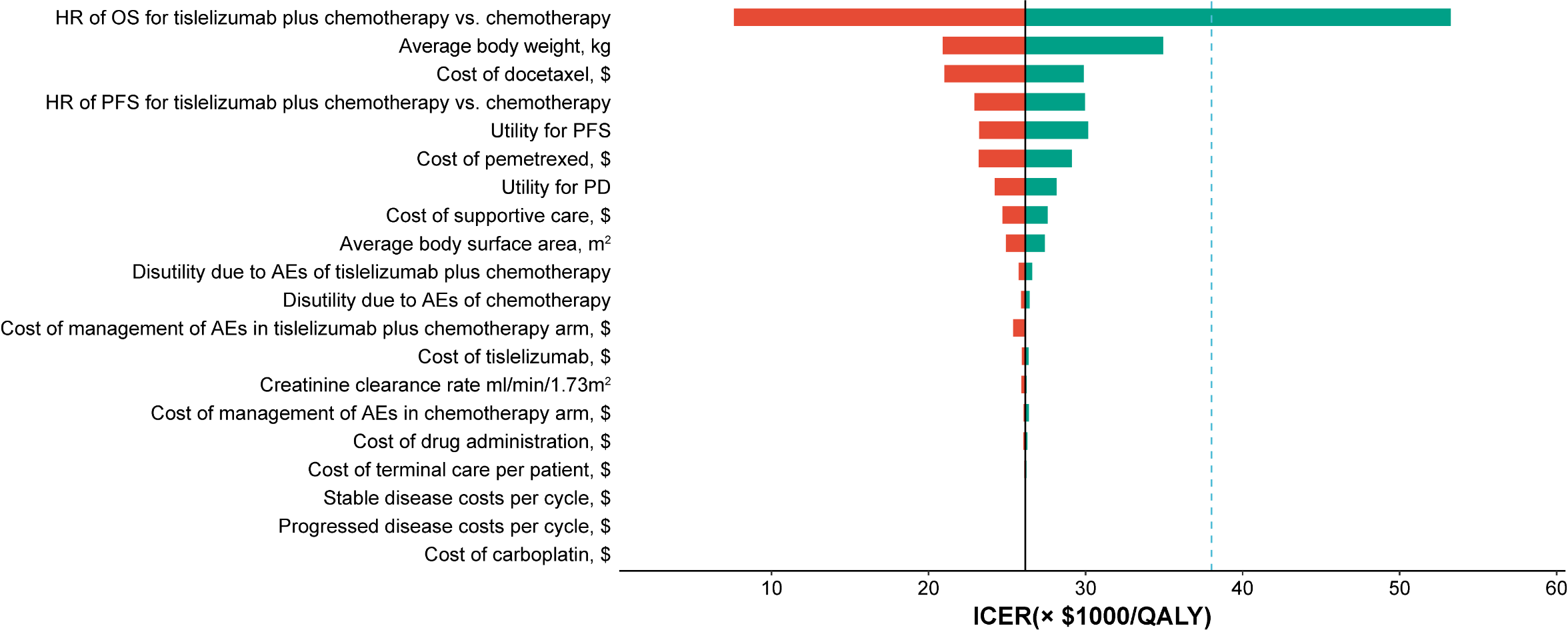


**Supplementary Figure 2.** Tornado Diagram of One-Way Sensitivity Analyses. OS, overall survival; HR, hazard ratio; PD, progressed disease; PFS, progression-free survival; AEs, adverse events.

**Supplementary Table 1.** Akaike Information Criterion and Bayesian Information Criterion Values from Each Survival Model

| **Strategies** | **Distributions** | **Parameters** | **mean** | **se** | **L95%** | **U95%** | **AIC** | **BIC** |
| --- | --- | --- | --- | --- | --- | --- | --- | --- |
| **Results of OS** | | | | | | | | |
| Tislelizumab plus chemotherapy | Exponential | rate | 0.00442315 | 0.0007175 | 0.00321846 | 0.00607875 | 489.989 | 493.396 |
|  | Weibull | shape | 1.923388409 | 0.2854128 | 1.43799 | 2.57263 | 476.808 | 483.622 |
|  |  | scale | 0.000140702 | 0.0001528 | 1.6749E-05 | 0.001182 |  |  |
|  | Gamma | shape | 2.3135138 | 0.451954 | 1.5775623 | 3.3927954 | 476.027 | 482.841 |
|  |  | rate | 0.0226193 | 0.0075305 | 0.0117786 | 0.0434375 |  |  |
|  | Lognormal | meanlog | 4.649847 | 0.172626 | 4.311507 | 4.98819 | **474.815** | **481.629** |
|  |  | sdlog | 0.972428 | 0.129116 | 0.749614 | 1.26147 |  |  |
|  | Gompertz | shape | 0.0383256 | 0.0119378 | 0.01492787 | 0.06172333 | 481.571 | 488.386 |
|  |  | rate | 0.00172924 | 0.0006441 | 0.00083334 | 0.00358829 |  |  |
|  | Log-logistic | shape | 2.04403 | 0.298841 | 1.53476 | 2.72229 | 476.268 | 483.082 |
|  |  | scale | 90.47498 | 13.136906 | 68.06668 | 120.26033 |  |  |
|  | Generalized gamma | mu | 4.584274 | 0.279118 | 4.037214 | 5.13134 | 476.557 | 486.779 |
|  |  | sigma | 1.197957 | 0.446694 | 0.576827 | 2.48793 |  |  |
|  |  | Q | -0.554434 | 1.180089 | -2.867367 | 1.7585 |  |  |
| Chemotherapy | Exponential | rate | 0.00719256 | 0.0013842 | 0.00493253 | 0.0104881 | 322.474 | 325.184 |
|  | Weibull | shape | 1.4391317 | 0.2505341 | 1.0231 | 2.024341 | 320.672 | 326.091 |
|  |  | scale | 0.0014457 | 0.0013616 | 2.28E-04 | 0.00915708 |  |  |
|  | Gamma | shape | 1.6366625 | 0.3695194 | 1.0514246 | 2.5476522 | 320.124 | 325.543 |
|  |  | rate | 0.0178408 | 0.0071592 | 0.0081253 | 0.0391731 |  |  |
|  | Lognormal | meanlog | 4.44785 | 0.222385 | 4.011987 | 4.88372 | **318.296** | **323.715** |
|  |  | sdlog | 1.17556 | 0.182503 | 0.867162 | 1.59365 |  |  |
|  | Gompertz | shape | 0.01623881 | 0.0140486 | -0.011296 | 0.0437736 | 323.164 | 328.583 |
|  |  | rate | 0.00508514 | 0.0019032 | 0.00244194 | 0.0105894 |  |  |
|  | Log-logistic | shape | 1.57856 | 0.268696 | 1.13076 | 2.20368 | 319.872 | 325.291 |
|  |  | scale | 77.94421 | 15.068931 | 53.36068 | 113.85351 |  |  |
|  | Generalized gamma | mu | 4.08847 | 0.594346 | 2.92357 | 5.25337 | 319.507 | 327.635 |
|  |  | sigma | 1.51641 | 0.306079 | 1.02096 | 2.25231 |  |  |
|  |  | Q | -1.11657 | 1.309106 | -3.68237 | 1.44923 |  |  |
| **Results of PFS** | | | | | | | | |
| Tislelizumab plus chemotherapy | Exponential | rate | 0.0164277 | 0.0016594 | 0.0134769 | 0.0200244 | 1003.323 | 1006.73 |
|  | Weibull | shape | 1.50791026 | 0.1303161 | 1.27295554 | 1.78623156 | **986.248** | **993.062** |
|  |  | scale | 0.00278133 | 0.0013145 | 0.00110143 | 0.00702338 |  |  |
|  | Gamma | shape | 1.7549218 | 0.2129917 | 1.3834038 | 2.2262124 | 986.266 | 993.08 |
|  |  | rate | 0.0369702 | 0.0065912 | 0.0260673 | 0.0524333 |  |  |
|  | Lognormal | meanlog | 3.68068 | 0.0918796 | 3.500595 | 3.86076 | 990.05 | 996.864 |
|  |  | sdlog | 1.03679 | 0.0788979 | 0.893134 | 1.20356 |  |  |
|  | Gompertz | shape | 0.0299554 | 0.0075902 | 0.01507899 | 0.0448319 | 989.968 | 996.782 |
|  |  | rate | 0.0091641 | 0.0017953 | 0.00624213 | 0.0134539 |  |  |
|  | Log-logistic | shape | 1.76313 | 0.150815 | 1.49098 | 2.08494 | 987.956 | 994.771 |
|  |  | scale | 39.0326 | 3.138432 | 33.34159 | 45.695 |  |  |
|  | Generalized gamma | mu | 3.882349 | 0.119463 | 3.648206 | 4.11649 | 988.201 | 998.423 |
|  |  | sigma | 0.706092 | 0.201637 | 0.403446 | 1.23577 |  |  |
|  |  | Q | 0.881047 | 0.522729 | -0.143483 | 1.90558 |  |  |
| Chemotherapy | Exponential | rate | 0.0244721 | 0.0033615 | 0.0186961 | 0.0320327 | 501.283 | 503.993 |
|  | Weibull | shape | 1.52058629 | 0.1662057 | 1.22735897 | 1.8838683 | **491.083** | **496.502** |
|  |  | scale | 0.00441424 | 0.0025412 | 0.00142834 | 0.0136421 |  |  |
|  | Gamma | shape | 1.8215209 | 0.293484 | 1.3282757 | 2.4979292 | 491.156 | 496.575 |
|  |  | rate | 0.0548962 | 0.012376 | 0.0352893 | 0.0853966 |  |  |
|  | Lognormal | meanlog | 3.280398 | 0.1160276 | 3.052988 | 3.50781 | 493.265 | 498.684 |
|  |  | sdlog | 0.958936 | 0.0949861 | 0.789724 | 1.16441 |  |  |
|  | Gompertz | shape | 0.0353489 | 0.0106272 | 0.01451993 | 0.0561778 | 493.074 | 498.493 |
|  |  | rate | 0.0135387 | 0.0033628 | 0.00832057 | 0.0220293 |  |  |
|  | Log-logistic | shape | 1.8361 | 0.20376 | 1.47719 | 2.28223 | 493.716 | 499.135 |
|  |  | scale | 27.1194 | 2.93297 | 21.93931 | 33.52263 |  |  |
|  | Generalized gamma | mu | 3.553894 | 0.220025 | 3.122653 | 3.98514 | 493.079 | 501.207 |
|  |  | sigma | 0.673586 | 0.264097 | 0.312361 | 1.45254 |  |  |
|  |  | Q | 0.948304 | 0.817315 | -0.653605 | 2.55021 |  |  |

**Supplementary Table 2.** Associated Costs and Disutility of Grade ≥ 3 Treatment-Related Adverse Events

| **Adverse Event** | **No. of patients (%)** | **Costs in 2021 USD^c^** | **Reference** | **Disutility** | **Reference** |
| --- | --- | --- | --- | --- | --- |
| **Tislelizumab plus chemotherapy** | |  |  |  |  |
| Anaemia | 33 (15%) | 3,667.90 | (1) | 0.072 | (2) |
| Neutrophil count decreased | 99 (45%) | 3,183.70 | (1) | 0.348 | (3) |
| White blood cell count decreased | 48 (22%) | 4714 | (4) | 0.072 | (3) |
| Platelet count decreased | 43 (19%) | 6928 | (5) | 0.108 | (6) |
| **Total** |  | 4326 |  | 0.202 |  |
| **chemotherapy** |  |  |  |  |  |
| Anaemia | 13 (12%) | 3,667.90 | (1) | 0.072 | (2) |
| Neutrophil count decreased | 39 (35%) | 3,183.70 | (1) | 0.348 | (3) |
| White blood cell count decreased | 16 (15%) | 4714 | (4) | 0.072 | (3) |
| Platelet count decreased | 15 (14%) | 6928 | (5) | 0.108 | (6) |
| **Total** |  | 3193 |  | 0.157 |  |

^a^Our analysis only included and evaluated grade ≥ 3 treatment-related adverse events.

^b^Number within treatment arm: tislelizumab plus chemotherapy (N=222), chemotherapy (N=110).

^c^Calculated as an average cost of toxicity using the weighted frequency of occurrence. This value was used in the base-case model.

**References**

1. Wu B, Dong B, Xu Y, Zhang Q, Shen J, Chen H, et al. Economic Evaluation of First-Line Treatments for Metastatic Renal Cell Carcinoma: A Cost-Effectiveness Analysis in a Health Resource-Limited Setting. *PLoS One* (2012) 7(3):e32530. doi: 10.1371/journal.pone.0032530

2. Freeman K, Connock M, Cummins E, Gurung T, Taylor-Phillips S, Court R, et al. Fluorouracil Plasma Monitoring: Systematic Review and Economic Evaluation of the My5-Fu Assay for Guiding Dose Adjustment in Patients Receiving Fluorouracil Chemotherapy by Continuous Infusion. *Health Technol Assess* (2015) 19(91):1-321, v-vi. doi: 10.3310/hta19910

3. Nafees B, Lloyd AJ, Dewilde S, Rajan N, Lorenzo M. Health State Utilities in Non-Small Cell Lung Cancer: An International Study. *Asia Pac J Clin Oncol* (2017) 13(5):e195-e203. doi: 10.1111/ajco.12477

4. Wong W, Yim YM, Kim A, Cloutier M, Gauthier-Loiselle M, Gagnon-Sanschagrin P, et al. Assessment of Costs Associated with Adverse Events in Patients with Cancer. *PLoS One* (2018) 13(4):e0196007. doi: 10.1371/journal.pone.0196007

5. Zheng H, Xie L, Zhan M, Wen F, Xu T, Li Q. Cost-Effectiveness Analysis of the Addition of Bevacizumab to Chemotherapy as Induction and Maintenance Therapy for Metastatic Non-Squamous Non-Small-Cell Lung Cancer. *Clin Transl Oncol* (2018) 20(3):286-93. doi: 10.1007/s12094-017-1715-1

6. Konidaris G, Paul E, Kuznik A, Keeping S, Chen CI, Sasane M, et al. Assessing the Value of Cemiplimab for Adults with Advanced Cutaneous Squamous Cell Carcinoma: A Cost-Effectiveness Analysis. *Value Health* (2021) 24(3):377-87. doi: 10.1016/j.jval.2020.09.014
